# Left Ventricular Blood Flow Kinetic Energy Assessment by 4D Flow Cardiovascular Magnetic Resonance: A Systematic Review of the Clinical Relevance †

**DOI:** 10.3390/jcdd7030037

**Published:** 2020-09-10

**Authors:** Harjinder Kaur, Hosamadin Assadi, Samer Alabed, Donnie Cameron, Vassilios S. Vassiliou, Jos J. M. Westenberg, Rob van der Geest, Liang Zhong, Amardeep Dastidar, Andrew J. Swift, Pankaj Garg

**Affiliations:** 1 Sheffield Teaching Hospital NHS Foundation Trust, Sheffield S10 2JF, UK; doctorgarcha@gmail.com (H.K.); S.Alabed@sheffield.ac.uk (S.A.); A.J.Swift@sheffield.ac.uk (A.J.S.); 2 Department of Infection, Immunity & Cardiovascular Disease, University of Sheffield, Sheffield S10 2JF, UK; HSAssadi1@sheffield.ac.uk; 3 Norwich Medical School, University of East Anglia, Norwich NR4 7TJ, UK; Donnie.Cameron@uea.ac.uk (D.C.); V.Vassiliou@uea.ac.uk (V.S.V.); 4 Radiology, Leiden University Medical Centre, 2333 ZA Leiden, The Netherlands; j.j.m.westenberg@lumc.nl (J.J.M.W.); R.J.van_der_Geest@lumc.nl (R.v.d.G.); 5 National Heart Centre Singapore, Duke-NUS Medical School, National University of Singapore, Singapore 169609, Singapore; zhong.liang@nhcs.com.sg; 6 Bristol Heart Institute, Bristol BS2 8ED, UK; dramar_deep@yahoo.co.uk

**Keywords:** intracardiac, systematic review, four-dimensional, 4D flow CMR, 4D flow MRI, cardiovascular magnetic resonance, phase contrast, velocity encoded, time-resolved

## Abstract

**Background:** There is an emerging body of evidence that supports the potential clinical value of left ventricular (LV) intracavity blood flow kinetic energy (KE) assessment using four-dimensional flow cardiovascular magnetic resonance imaging (4D flow CMR). The aim of this systematic review is to summarize studies evaluating LV intracavity blood flow KE quantification methods and its potential clinical significance. **Methods:** A systematic review search was carried out on Medline, Pubmed, EMBASE and CINAHL. **Results:** Of the 677 articles screened, 16 studies met eligibility. These included six (37%) studies on LV diastolic function, another six (37%) studies on heart failure or cardiomyopathies, three (19%) studies on ischemic heart disease or myocardial infarction and finally, one (6%) study on valvular heart disease, namely, mitral regurgitation. One of the main strengths identified by these studies is high reproducibility of LV blood flow KE hemodynamic assessment (mean coefficient of variability = 6 ±  2%) for the evaluation of LV diastolic function. **Conclusions:** The evidence gathered in this systematic review suggests that LV blood flow KE has great promise for LV hemodynamic assessment. Studies showed increased diagnostic confidence at no cost of additional time. Results were highly reproducible with low intraobserver variability.

## 1. Introduction

The hemodynamic loads exerted on the cardiovascular system—mainly the left ventricle (LV)—are the leading cause of cardiovascular diseases [1,2]. LV intracavity flow is generated by pressure gradients, which in turn, are the result of LV mechanical power [3]. The LV power can be described as an active contraction of the myocardium in systole and active relaxation during diastole. The direct assessment of the LV intracavity flow in three dimensions (3D) offers novel insight into complex flow patterns associated with LV circulation, both in health and disease states. A 3D flow assessment which can be investigated over the complete cardiac cycle is currently possible due to emerging methods of four-dimensional flow cardiovascular magnetic resonance (4D flow CMR) [4,5]. This is described as ‘four-dimensional’ because it encodes velocity in all three spatial directions, as well as time.

The LV intracavity flow patterns change during the cardiac cycle. For example, during diastolic filling, a 3D vortex ring is formed at the base of LV. This vortex ring characteristically adapts during the course of diastole [6]. All the energy in the vortex is then directed towards the outflow tract in systole. The complex patterns of the blood flow in the LV and their behavior is difficult to routinely quantify in clinical practice. For routine clinical translation, simplified methods have been proposed. One such method is to quantify the kinetic energy (KE) of the blood flow inside the LV during the whole cardiac cycle. From a technical perspective, this can be achieved by using the routinely done time-resolved, endocardial contours on short-axis LV cine stack for volumetric assessment. These endocardial contours can extract velocity data of the LV intracavity using the 4D flow CMR dataset. As this method allows instantaneous, quantitative assessment of LV intracavity flow, it has potential utility in a busy clinical environment. Several studies have investigated the role of LV intracavity blood flow KE as imaging biomarkers of LV hemodynamics.

The aim of this systematic review is to methodically summarize CMR studies evaluating LV intracavity blood flow KE quantification methods and its potential clinical significance.

## 2. Methods

### 2.1. Systematic Review Registration

This systematic review was prospectively registered (CRD42019117855) [7] with the international database of prospectively registered systematic reviews (PROSPERO), the international database of prospectively registered systematic reviews in health where there is a health-related outcome.

### 2.2. Search Strategy

**Figure 1:**
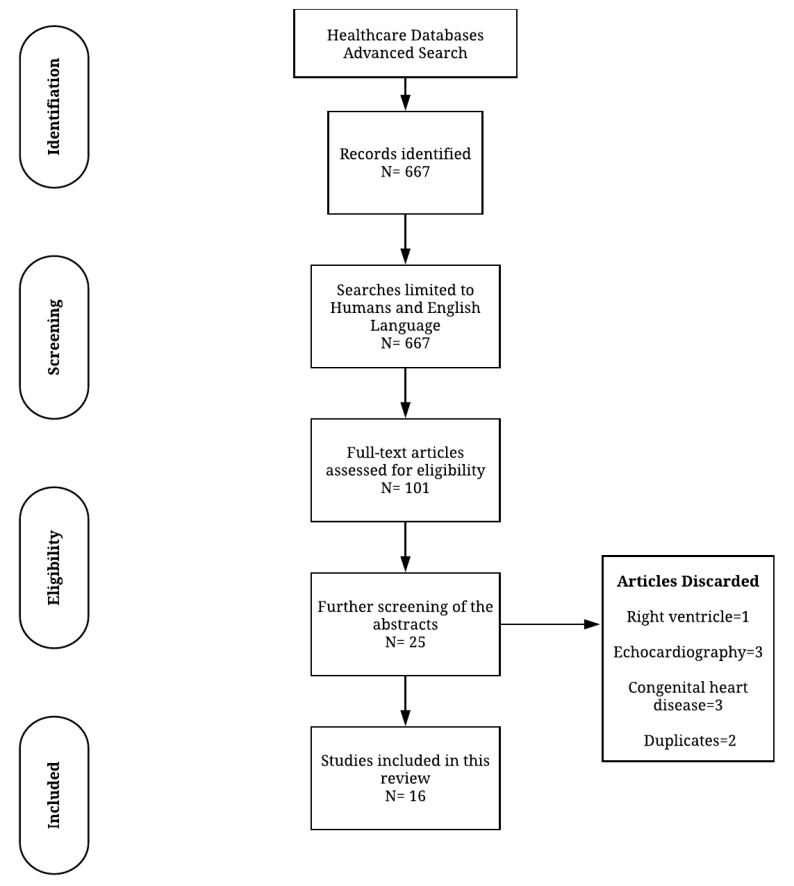
Flow diagram demonstrating evidence synthesis for the systematic review, adapted from Moher et al. 2009 [8] using the PRISMA tool.

**Figure 2:**
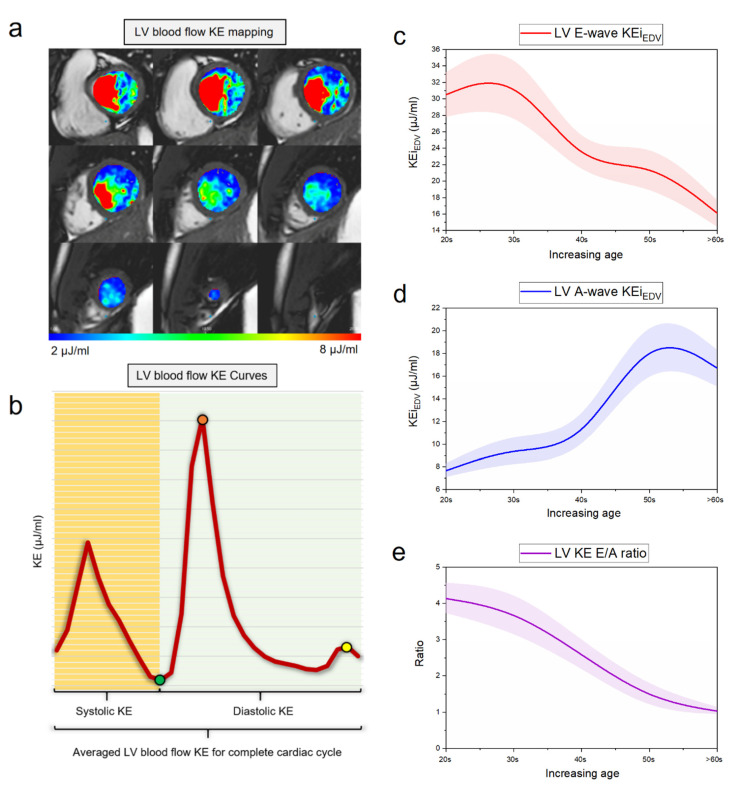
Left ventricular kinetic energy (KE) mapping. (**a**) Mapping of left ventricular (LV) KE using endocardial contours. This is a semi-automated method for the quantification of hemodynamically relevant parameters; (**b**) left ventricular blood flow KE curve for the whole cardiac cycle; green point: end-systolic KE; yellow point: Peak A-wave KE; red point: Peak E-wave KE; (**c**) mean line of LV E-wave KE with progressive age demonstrates that with age E-wave KE reduces; (**d**) mean line of LV A-wave KE with progressive age demonstrates that with age E-wave KE increases; (**e**) mean line of LV KE ratio with progressive age demonstrates that with age KE E/A ratio reduces.

**Figure 3:**
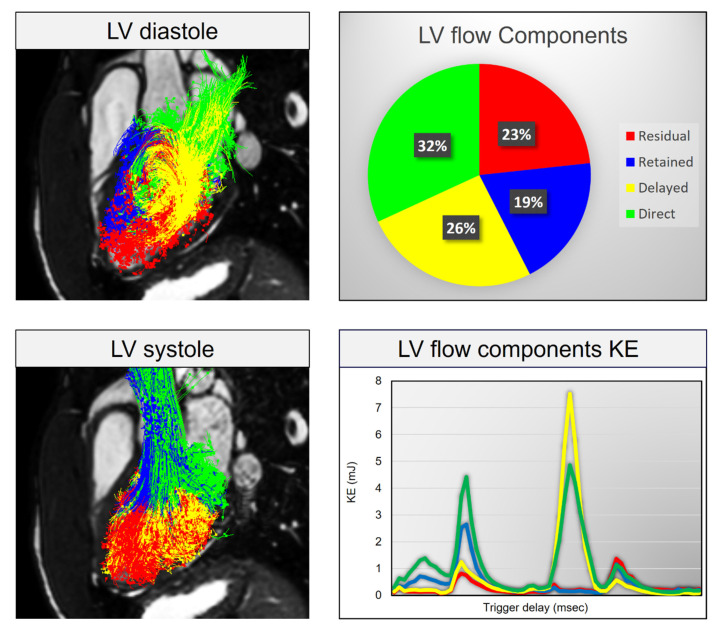
LV blood-flow components KE analysis. LV blood-flow component KE breakdown can give further insight into kinetic energy distribution between different flow components in various cardiovascular disorders. Red: Residual flow component, Blue: Retained flow component, Yellow: Delayed flow component, Green: Direct flow component.

A comprehensive search was undertaken on the 5th of January 2019. Several electronic databases (Medline, Pubmed, EMBASE and CINAHL) were searched individually using the healthcare databases advanced search (HDAS) provided by the National Institute of Clinical Excellence (NICE), United Kingdom (Figure 1). Our search strategy included the following terms: Left ventricle kinetic energy; Left ventricle energetics; Intra ventricular energetics; Four-dimensional flow energetics; 4D flow kinetic energy; 4D flow energetics; LV energetics and cardiac magnetic resonance flow kinetic (Figure 2 and Figure 3). All searches were combined and duplicates removed. As each database has a unique exclusion variable, all exclusion variables for individual database searches are listed in the Supplementary documents (Table S1) (Figure S1) (Figure S2).

The Supplemental Material provides an in-depth description of the search strategy. Citation tracking and manual reference searching were carried out through the HDAS databases. The preferred reporting items for systematic reviews and meta-analysis (PRISMA) checklist was followed when structuring this article (Figure 1) [8].

### 2.3. Article Screening

A total of 667 studies were initially identified with the search strategy. After an initial screening by HK, 101 studies were shortlisted and included. Article abstracts were reviewed further by PG to exclude irrelevant studies. This narrowed the screened articles to 25 after further discussions among the three reviewers. Cases of disagreement were discussed between the reviewers to reach a suitable conclusion. The studies that passed this screening, had their full texts evaluated.

A meta-analysis was deemed inappropriate for this systematic review as much of the research is exploratory with considerable heterogeneity in the included studies. As a result, a narrative review is provided.

## 3. Results

After a comprehensive review, 25 studies were considered suitable for the systematic review. Further nine studies (1, 2, 5, 7, 10, 11, 14, 21, 24) were excluded after having read through the paper carefully. The reason for exclusion was the following: right ventricle (1), used echocardiography (2, 10, 14), focused on congenital heart disease (5, 7, 24) and duplicates (11, 21).

The 16 remaining studies were further categorized into six (37%) studies on the topic of LV diastolic function and aging, another six (37%) studies on heart failure (HF) including cardiomyopathies, three (19%) studies on ischemic heart disease or myocardial infarction and finally, one (6%) study on valvular heart disease, namely mitral regurgitation (MR).

Of all studies, five [6,9,10,11,12] commented about the reproducibility. Further details of individual papers are highlighted in the discussion section and a detailed breakdown for each study is provided in the Supplemental Material.

## 4. Discussion

In this systematic review, the evidence from the pooled studies highlight that LV blood flow kinetic energy (KE) assessment by 4D flow CMR is an emerging novel technique for the assessment of cardiovascular hemodynamics. LV energetics assessment has demonstrated clinical utility in LV diastolic assessment (Figure 2), prediction of LV thrombus (LVT) formation post-myocardial infarction (MI), predicting adverse LV remodeling post-MI for further risk stratification of patients and finally, in both ischemic/nonischemic cardiomyopathies (Figure 3). In addition, all global KE parameters demonstrated excellent intraclass correlation coefficient (ICC) (average 0.99, *p* > 0.9) which is important in clinical translation of this novel imaging biomarker for hemodynamic assessment [10]. The characteristics of studies which were included in this systematic review are shown in (Table 1).

### 4.1. Aging and LV Diastolic Function

Four-dimensional flow cardiac magnetic resonance proved to provide consistent information on the decline in early diastolic kinetic energy with aging [13]. Four-dimensional CMR provides a detailed overview of the velocity of blood in three directions which helps to capture intricacies of blood flow within the heart. Furthermore, CMR has suggested a decrease in the number of vortices with age, affecting the flux of energy [14] which leads to loss in blood momentum, thus, contributing to heart failure (HF) [15]. Previous literature suggests progressive and significant fall in mean early diastolic KE peaks after the age of 40 years. Reduced early diastolic KE in healthy old adults are similar to those values seen in HF patients [16]. This inconsistency may be due to a decrease in ventricular compliance leading to less favorable LV filling and impaired suction of blood from left atrium and pulmonary veins [12]. Further, changes in LV morphology, increased LV stiffness due to accumulation of collagen and increased extracellular matrix cross-linking leading to decreased compliance thus contributing to diastolic dysfunction [17]. These findings are consistent with that of Wong et al. who compared intraventricular KE in a healthy group of various ages with subjects with LV dysfunction. They recruited 45 individuals, 35 healthy (78%) (1 to 67 years of age), divided into four age quartiles (eight children, 11 young adults, 11 middle-aged adults and eight older adults) and 10 (22%) with compensated LV dysfunction (28–79 years of age). With the help of 4D flow MRI, they demonstrated that peak diastolic KE progressively decreased in the 1st and 2nd age quartiles (*p* < 0.007) and a second dip was observed between 2nd and 4th age quartiles (*p* = 0.025). Four-dimensional flow CMR is capable of capturing intricacies of blood flow within the heart in three directions whereas two-dimensional (2D) echocardiography and 2D CMR could measure it in one direction only. Compared with children and young adults, older adults had a lower early peak diastolic KE (*p* < 0.0001 and *p* = 0.025) respectively. In another study, Crandon et al. [9] suggested that 4D CMR biomarkers of LV blood flow KE showed stronger association with age than the corresponding 2D metrics, peak E-wave and A-wave velocities (r = −0.51 vs. −0.17 and r = 0.65 vs. 0.46). Fifty-three healthy volunteers underwent standard CMR and 4D flow acquisition. LV blood flow KE parameters demonstrated good reproducibility with mean coefficient of variation of 6 ± 2% with accuracy and precision of (99%, 97%) respectively. There was a good association between the LV blood flow kinetic energy indexed to end-diastolic volume (KE_iEDV_) E/A ratio and 2D mitral inflow E/A ratio (r = 0.77, *p* < 0.01) decreasing progressively with age (*p* < 0.01). Another important finding was observed by Zajac et al. [18]. They assessed and compared turbulent kinetic energy (TKE) values within the left ventricle of 20 subjects (11 healthy and nine dilated cardiomyopathy [DCM] patients) using novel 4D flow CMR. This was based on when blood flow becomes turbulent, TKE is transferred from the mean flow to small turbulent eddies where viscous forces dominate and the energy is dissipated into heat, which can be seen as a measure of flow inefficiency. The study compared peak TKE at early and late diastolic filling and correlated with mitral annular dimensions in healthy & DCM subjects. It concluded no differences in early diastolic filling, but TKE at late diastolic filling was higher in DCM patients (*p* < 0.001) and correlated with LV diameter (*p* = 0.02) and transmitral velocity (*p* < 0.01). Thus, TKE measured with CMR in decompensated DCM patients by late diastolic LV TKE values exceed those with normal LV. On the other hand, Steding-Ehrenborg et al. [19] demonstrated that LV early diastolic KE is strongly associated with LV mass (LVM). Twenty-eight individuals (14 athletes and 14 sedentary volunteers) underwent 4D phase contrast CMR for KE quantification in four chambers and energy expenditure estimation using the mean kinetic energy/cardiac index (KE/CI) parameter. Athletes have higher KE in early diastole due to high LVM leading to enhanced diastolic function. Among the various filling mechanisms for the ventricles, LVM was a determinant factor of kinetic energy for LV filling as shown in athletes and sedentary individuals (r² = 0.66 *p* < 0.001). Increased LV mass in athletes is not pathologic due to preserved geometry (athletes have thicker LV wall and may present with concentric hypertrophy based on the ratio between wall thickness and ventricular diameter). However, this is called ‘physiological hypertrophy’ as the heart adapts to long term endurance. The peak KE at early diastole in LV is higher in athletes (8.9 ± 1.1 vs. 5.9 ± 0.4 mJ, *p* = 0.004) while the mean KE of LV over the cardiac cycle remains the same and the peak oxygen uptake (VO2max) correlated with mean KE (LVKE: r² = 0.37, *p* < 0.05). In addition, the amount of KE needed to meet the metabolic demands at rest, measured as mean LVKE/CI (as indexed by cardiac index) did not differ between athletes and sedentary subjects (0.56 ± 0.06 and 0.53 ± 0.04 mJ·L^−^¹.min.m², *p* = 0.98), due to lower heart rate cancelling increased KE per beat in athletes. Measuring the amount of KE inside and outside the LV diastole vortex further contribute towards determining the kinetic energy blood flow in athletes and sedentary controls. The mean KE of the diastolic vortex contributed (70 ± 1% and 73 ± 2%, *p* = 0.18) of LV total diastolic KE in athletes and controls, respectively. Moreover, the percentage of ventricular KE made up by vortex KE showed no differences between athletes and controls (early diastole: 67 ± 1% vs. 71 ± 2%, *p* = 0.17; diastasis: 73 ± 2% vs. 70 ± 6%, *p* = 0.78; and late diastole: 73 ± 2% vs. 71 ± 6%, *p* = 0.29).

In a study by Carlsson et al. [12] nine healthy volunteers with no history of cardiovascular or systemic disease underwent four-dimensional postcontrast CMR for KE quantification of LV during the entire cardiac cycle using in-house developed software. Mean KE was related to end-diastolic volume (EDV), end-systolic volume (ESV) and stroke volume (SV) for LV (r² = 0.66, r² = 0.59 and r² = 0.55). There were three KE peaks in systole, early and late diastole (4.9 ± 0.4 mJ; 6.0 ± 0.6 mJ and 1.3 ± 0.2 mJ, *p* = 0.004, respectively) LV KE during early diastole was higher in LV than (6 ± 0.6 mJ, *p* = 0.004). High early diastolic KE in the LV indicates that LV filling is more dependent on ventricular suction as a result of wall tension.

### 4.2. Ischemic Heart Disease and Myocardial Infarction

Garg et al. [10] studied LV flow kinetics and infarct characterization in 48 patients with acute and chronic MI and demonstrated that intracavity blood flow kinetic energy is altered in heart failure patients—even if the LV ejection fraction (EF) is preserved. This has been identified with three blood flow novel KE parameters: the proportion of in-plane LV KE; time difference (TD) of peak E-wave KE and minimal KE_iEDV_. The results obtained from patients who completed full study showed infarct size was similar in acute and chronic MI patients and the majority had anterior MI. The averaged KE_iEDV_ and minimal LV flow KE_iEDV_ were significantly lower in MI patients (*p* = 0.02). Similarly, diastolic peak E-wave KE_iEDV_ was significantly lower in MI patients (*p* < 0.005) and the time difference to peak E-wave KE_iEDV_ was significantly higher in MI group (*p* < 0.01). Also, diastolic peak E-wave KE_iEDV_ was significantly lower in MI (*p* < 0.005) and several LV flow KE_iEDV_ demonstrated significant differences between LV systolic impairment groups of patients (*p* < 0.05). In another study, Garg et al. [11] compared LV blood flow KE using 4D flow CMR in myocardial infarction patients with and without LV thrombus. They conducted a prospective cohort study of 108 subjects (40 controls, 36 MI with no thrombus and 32 MI with thrombus) from two centers—Leeds and Leiden. Due to the recent development in mapping and quantification of intracavity LV flow and its potential to provide new mechanistic insights into the pathophysiology of LV thrombus, 4D flow CMR mapped LV flow KE and characterized flow changes in patients with ischemic cardiomyopathy with LV thrombus and without LV thrombus. KE mapping suggested a higher proportion of in-plane KE in patients with LV thrombus vs. without LV thrombus (*p* = 0.002), whereas peak late filling KE in end-diastole volume was not different between the three groups (*p* > 0.05). Patients with LV thrombus demonstrated a significantly higher drop in peak late filling KE from mid to apex compared to LV without thrombus patients (*p* < 0.01). It is also suggested that during early and late diastole, blood flow into the LV cavity takes very little time due to intraventricular pressure gradients. Increasing time difference from healthy controls to LV without thrombus and further to LV with thrombus patients demonstrated that patients with LV thrombus have a significantly delayed wash-in of the LV. These data also supported that increase in the in-plane flow will reduce the proportion of through-plane flow in the LV cavity and thus less blood will pass through the ventricle per unit time resulting in reduced global wash-in and wash-out of the LV. Another important finding was that of Chan et al. who suggested that vortex KE can be used as an early predictor of LV progressive dysfunction overflow energetic indices e.g., KE fluctuations and energy dissipation index (DI) [20]. Chan et al. is the first study to investigate the intraventricular flow–energetic indices in 20 healthy participants and 30 ischemic heart disease (IHD) patients (14 with normal EF and 16 with reduced EF) using postcontrast CMR. The key findings were that the healthy and normal EF group had a high KE fluctuation index concentrated near the LV outflow tract (LVOT). There were no differences in intraventricular flow variables between the healthy and the normal ejection fraction group in comparison with the reduced EF group in which there was a considerable reduction in KE fluctuation index (*p* < 0.001) and vortex KE (*p* = 0.003). Furthermore, the magnitude and spatial distribution of flow–energetic indices were also quite similar in both the healthy and preserved normal EF group in contrast to the reduced EF group. This is largely explained due to the increased KE fluctuation index inside the vortex than outside in the healthy and the preserved EF group.

### 4.3. Heart Failure Including Cardiomyopathies

Direct flow volume and KE diminishes with increased LV volumes. Svalbring et al. suggested in his paper that subtle or subclinical LV remodeling can be detected by 4D flow CMR particularly in the direct flow phase [21]. Thirty-six (26 heart failure patients and 10 healthy controls) underwent CMR for LV KE assessment. The study hypothesized that all the four components in a normal heart would have a consistent distribution of component volumes, routes and energetics. The direct flow appeared to have the most direct route, fastest transit through LV and best preserves the kinetic energy from inflow to presystole. Direct flow volume and KE decreased with increasing LVEDV-index (LVEDVI) and LVESV-index (LVESVI) (direct flow volume r = −0.64 and r = −0.74, both *p* < 0.001; direct flow KE r = −0.48, *p* = 0.013 and r = −0.56, *p* = 0.003). KE and non-ejecting flow volume increased with rising LVEDVI and LVESVI (non-ejecting flow KE: r = 0.53, *p* = 0.005 and r = 0.52, *p* = 0.006, both *p* < 0.001; non-ejecting flow volume: r = 0.67 and r = 0.76). These results reflect those of Kanski et al. who also investigated HF patients. By looking into 39 patients (29 HF and 12 health controls), they assessed LV KE in heart failure patients and the majority (79%) had left bundle branch block (LBBB) with IHD as the most common etiology. KE was calculated within the whole LV and vortex ring. The study exhibited indexed KE to SV and showed that HF patients compared to controls had higher values for both the systolic average KE/SV (28.3 ± 18.4 μJ/mL vs. 12.9 ± 2.9 μJ/mL, *p* < 0.0001) and diastolic average KE/SV (40.8 ± 29.7 μJ/mL vs. 16.3 ± 4.1 μJ/mL, *p* < 0.0001). However, when indexed to EDV, HF patients showed lower systolic average KE compared to controls (6.3 ± 2.2 μJ/mL vs. 8.0 ± 2.1 μJ/mL, *p* = 0.025) with no difference in diastolic average KE (9.0 ± 4.4 μJ/mL vs. 10.2 ± 3.3 μJ/mL, *p* = 0.41). There were no statistically significant differences in systolic and diastolic KE peak value between heart failure patients and controls (*p* = 0.92, *p* = 0.29). The most important point highlighted in this study is that despite the dyssynchrony affecting KE, regional wall motion in the presence or absence of septal dyskinesia had no impact on KE in this study. Another study on HF patients was done by Zajac et al. who sought to investigate the impact of mechanical dyssynchrony on diastolic function by comparing 4D flow in myopathic LVs with and without LBBB [22]. They recruited 22 HF patients (half of them with LBBB). After 4D flow CMR analysis, KE at end-diastole and the direct flow entering the LV during early diastolic filling showed less KE at end-diastole in LBBB patients compared to non LBBB patients (*p* = 0.018). LV Direct Flow KE in presystole was less in patients with LBBB compared to non LBBB patients. Therefore, 4D flow CMR can help as a marker of LV mechanical dyssynchrony in HF. Eriksson et al. demonstrated that HF patients with mild LV remodeling demonstrate altered diastolic flow routes through the LV and impaired preservation of inflow KE at presystole compared with healthy subjects [23]. Twenty individuals (10 with DCM and 10 healthy) underwent 4D CMR for acquiring velocity and morphologic data. In DCM patients, EDV was larger (*p* = 0.021) and EF was smaller (*p* < 0.001) compared with healthy subjects; the SV was equivalent (DCM: 77 ± 19 mL, healthy: 79 ± 16 mL) and total LV inflow that passed directly to ejection was smaller in DCM (*p* < 0.01). However, end-diastolic KE per mL of blood of the direct flow was similar in the two groups. Bolger et al. study, on the other hand, performed CMR 4D flow phase contrast velocity mapping in 17 normal subjects including one with DCM. They measured the relative volume of LV flow components and diastolic changes in inflowing KE. They eluded that in healthy subjects, 16 ± 8% of KE of inflow was conserved to the end of diastole compared with 5% in DCM. The reduction in KE of retained inflow in DCM patients was 18-fold greater than that of blood tracing the direct route. Four-dimensional CMR was used in order to assess LV diastolic function by various methods—phase contrast CMR data acquisition; flow separation and visualization; flow quantification and statistical analysis. Four components (direct flow; retained flow; delayed ejection and residual volume flow) of LV flow were measured in normal subjects against myopathic LV. The original proportions and routes of the inflow components noted to be varied with the phase in the cardiac cycle and vortical flow noted on the ventricular side of the mitral leaflets and LV apical area in all the subjects. The direct flow path consistently runs from the basal half of LV around the edge of the anterior leaflet and then flows toward LV outflow tract, while other flow components run along other regions of LV.

### 4.4. Valvular Heart Disease

In a study done by Al-Wakeel et al. [24], intracardiac blood flow quantification by KE was altered in patients with mitral regurgitation (MR). They compared the results of pre (6 patients) and post mitral valve (MV) surgery (4 patients) with seven healthy volunteers. EDV, ESV and SV were significantly higher in patients with MR, pre and postoperatively than in healthy volunteers (all *p* < 0.01). There was a significant decrease in mean KE, systolic and early diastolic KE peaks postoperatively (all *p* < 0.05). However, not much change in late diastolic peak KE was observed. The most important finding of their study was that physiological flow conditions did not fully normalize post mitral valve surgery—highlighting much more needs to be done to improve therapeutic interventions in valvular heart disease.

### 4.5. Clinical Perspective

In this review, we highlight some of the clinical studies demonstrating mechanistic insight into how LV blood flow KE assessment can offer enhanced and reliable assessment of LV hemodynamics. However, more work needs to be done to establish patho–physiological cascade of LV blood-flow behavior from healthy hearts to disease state. Rise in intracardiac pressure is the hallmark of heart failure. Future studies should also explore the relationship of LV blood flow KE with intracavity pressure throughout the cardiac cycle. This will help develop superior noninvasive LV KE biomarkers of intracardiac pressures which can be applied in clinical setting to inform diagnosis and possibly optimize treatment to improve clinical outcomes in heart disease. LV blood KE in particular may have a role in aortic regurgitation assessment as aortic valve incompetency mainly results in a jet inside the LV cavity. Future studies are needed to evaluate how LV blood flow KE changes in aortic regurgitation and could this be used for informing timing of intervention. Even though aortic stenosis causes blood flow acceleration in out flow tract predominantly, it is affect on the LV blood flow KE remains unknown. To summarize, LV blood flow KE assessment not only has the potential to improve diagnostic strategies for LV diastolic function, but also in many other cardiovascular disorders including valvular heart disease.

## 5. Conclusions

The evidence gathered in this systematic review suggests that LV blood flow KE holds great promise for LV hemodynamic assessment. Studies showed increased diagnostic confidence at no cost of additional time. Results were highly reproducible with a low intraobserver variability. However, the prognostic role of these novel imaging-based biomarkers of flow remains undetermined. Future clinical studies are warranted to evaluate the prognostic role of using these novel intraventricular flow biomarkers.

## Figures and Tables

**Table 1:** Characteristics of studies included in this systematic review.

	First Author	Year	Method	N	Reproducibility	Disease
**Aging and LV diastolic function**						
1	Wong et al.	2016	LV KE	45	−	Aging and LV diastolic assessment
2	Crandon et al.	2018	LV KE	53	+	Aging and LV diastolic assessment
3	Zajac et el.	2015	LV TKE	20	−	LV diastolic assessment
4	Steding-Ehrenborg et al.	2015	LV KE	28	−	Athletes vs. sedentary
5	Carlsson et al.	2011	LV KE	9	+	Healthy—mechanistic
6	Kim et al.	1995	**Vortex**	26	+	Healthy—mechanistic
**Ischemic heart disease and myocardial infarction**						
7	Garg et al.	2018	LV KE MI	58	+	MI
8	Garg et al.	2018	LV KE thrombus	108	+	LV thrombus
9	Chan et al.	2018	LV KE and vortex	50	−	STEMI
**Heart failure including cardiomyopathies**						
10	Svalbring et al.	2016	LV DF KE	36	−	HFrEF
11	Eriksson et al.	2012	LV PT + KE	20	−	DCM
12	Kanski et al.	2015	LV KE	41	−	HFrEF
13	Zajac et al.	2017	LV PT + KE	22	−	HF with LBBB
14	Eriksson et al.	2011	LV PT + KE	13	−	DCM
15	Bolger et al.	2007	LV PT + KE	18	−	DCM
**Valvular heart disease**						
16	Al-Wakeel et al.	2015	LV KE in MR	17	−	MV—mitral regurgitation

**Abbreviations:** DCM—dilated cardiomyopathy; DF—diastolic function; HF—heart failure; HFrEF—heart failure with reduced ejection fraction; KE—kinetic energy; LBBB—left bundle branch block; LV—left ventricle; MI—myocardial infarction; MR—mitral regurgitation; MV—mitral valve; PT—pacing threshold; STEMI—st-elevation myocardial infarction; TKE—turbulent kinetic energy. ‘−‘ no reproducibility data, ‘+’ reproducibility checked.

## Supplementary Materials

The following are available online at https://www.mdpi.com/2308-3425/7/3/37/s1.

## Abbreviations

2D	two-dimensional
3D	three-dimensional
4D	four-dimensional
CMR	cardiac magnetic resonance
DCM	dilated cardiomyopathy
DI	dissipation index
EDV	end-diastolic volume
ESV	end-systolic volume
EF	ejection fraction
HFrEF	heart failure with reduced ejection fraction
HDAS	healthcare databases advanced search
IHD	ischemic heart disease
KE	kinetic energy
KE/CI	mean kinetic energy/cardiac index
KE_iEDV_	kinetic energy indexed end-diastolic volume
LBBB	left bundle branch block
LV	left ventricle/left ventricular
LVEDV	left ventricular end-diastolic volume
LVEDVi	left ventricular end-diastolic volume index
LVESVi	left ventricular end-systolic volume index
LVM	left ventricular mass
LVOT	left ventricular outflow tract
MI	myocardial infarction
mJ	millijoules
MR	mitral regurgitation
MRI	magnetic resonance imaging
MV	mitral valve
PRISMA	preferred reporting items for systematic reviews and meta-analyses
PROSPERO	international prospective register of systematic reviews
RV	right ventricle
SV	stroke volume
TD	time difference
TKE	turbulent kinetic energy

## References

[1] Sengupta P.P., Pedrizzetti G., Kilner P.J., Kheradvar A., Ebbers T., Tonti G., Fraser A.G., Narula J. (2012). Emerging trends in CV flow visualization. JACC Cardiovasc. Imaging.

[2] Doost S.N., Ghista D., Su B., Zhong L., Morsi Y.S. (2016). Heart blood flow simulation: A perspective review. Biomed. Eng. Online.

[3] Borlaug B.A., Kass D.A. (2009). Invasive hemodynamic assessment in heart failure. Heart Fail Clin..

[4] Dyverfeldt P., Bissell M., Barker A.J., Bolger A.F., Carlhäll C.J., Ebbers T., Francios C.J., Frydrychowicz A., Geiger J., Giese D. (2015). 4D flow cardiovascular magnetic resonance consensus statement. J. Cardiovasc. Magn. Reason. J. Soc. Cardiovasc. Magn. Reson..

[5] Van der Geest R.J., Garg P. (2016). Advanced Analysis Techniques for Intra-cardiac Flow Evaluation from 4D Flow MRI. Curr. Radiol. Rep..

[6] Kim W.Y., Walker P.G., Pedersen E.M., Poulsen J.K., Oyre S., Houlind K., Yoganathan A.P. (1995). Left ventricular blood flow patterns in normal subjects: A quantitative analysis by three-dimensional magnetic resonance velocity mapping. J. Am. Coll. Cardiol..

[7] Harjinder K., Garg P. (2019). The Clinical Benefit of Four-Dimensional Cardiac Magnetic Resonance Derived Left Ventricle Energetics: A Systematic Review.

[8] Moher D., Liberati A., Tetzlaff J., Altman D.G., PRISMA Group (2009). Preferred reporting items for systematic reviews and meta-analyses: The PRISMA statement. PLoS Med..

[9] Crandon S., Westenberg J.J., Swoboda P.P., Fent G.J., Foley J.R., Chew P.G., Brown L.A., Saunderson C., Al-Mohammad A., Greenwood J.P. (2018). Impact of Age and Diastolic Function on Novel, 4D flow CMR Biomarkers of Left Ventricular Blood Flow Kinetic Energy. Sci. Rep..

[10] Garg P., Crandon S., Swoboda P.P., Fent G.J., Foley J.R., Chew P.G., Brown L.A., Vijayan S., Hassell M.E., Nijveldt R. (2018). Left ventricular blood flow kinetic energy after myocardial infarction—insights from 4D flow cardiovascular magnetic resonance. J. Cardiovasc. Magn. Reason. J. Soc. Cardiovasc. Magn. Reson..

[11] Garg P., Van Der Geest R.J., Swoboda P.P., Crandon S., Fent G.J., Foley J.R., Dobson L.E., Al Musa T., Onciul S., Vijayan S. (2018). Left ventricular thrombus formation in myocardial infarction is associated with altered left ventricular blood flow energetics. Eur. Heart J. Cardiovasc. Imaging.

[12] Carlsson M., Heiberg E., Toger J., Arheden H. (2012). Quantification of left and right ventricular kinetic energy using four-dimensional intracardiac magnetic resonance imaging flow measurements. Am. J. Physiol. Heart Circ. Physiol..

[13] AlGhatrif M., Strait J.B., Morrell C.H., Canepa M., Wright J., Elango P., Scuteri A., Najjar S.S., Ferrucci L., Lakatta E.G. (2013). Longitudinal trajectories of arterial stiffness and the role of blood pressure: The Baltimore Longitudinal Study of Aging. Hypertension.

[14] Föll D., Taeger S., Bode C., Jung B., Markl M. (2013). Age, gender, blood pressure, and ventricular geometry influence normal 3D blood flow characteristics in the left heart. Eur. Heart J. Cardiovasc. Imaging.

[15] Eriksson J., Bolger A.F., Ebbers T., Carlhäll C.-J. (2013). Four-dimensional blood flow-specific markers of LV dysfunction in dilated cardiomyopathy. Eur. Heart J. Cardiovasc. Imaging.

[16] Wong J., Chabiniok R., Devecchi A., Dedieu N., Sammut E., Schaeffter T., Razavi R. (2016). Age-related changes in intraventricular kinetic energy: A physiological or pathological adaptation?. Am. J. Physiol. Heart Circ. Physiol..

[17] De Souza R.R. (2002). Aging of myocardial collagen. Biogerontology.

[18] Zajac J., Eriksson J., Dyverfeldt P., Bolger A.F., Ebbers T., Carlhäll C.-J. (2015). Turbulent kinetic energy in normal and myopathic left ventricles. J. Magn. Reason. Imaging.

[19] Steding-Ehrenborg K., Arvidsson P.M., Töger J., Rydberg M., Heiberg E., Carlsson M., Arheden H. (2016). Determinants of kinetic energy of blood flow in the four-chambered heart in athletes and sedentary controls. Am. J. Physiol. Heart Circ. Physiol..

[20] Chan B.T., Yeoh H.K., Liew Y.M., Dokos S., Al Abed A., Chee K.H., Abdul Aziz Y.F., Sridhar G.S., Chinna K., Lim E. (2018). Quantitative analysis of intraventricular flow-energetics and vortex in ischaemic hearts. Coron. Artery Dis..

[21] Svalbring E., Fredriksson A., Eriksson J., Dyverfeldt P., Ebbers T., Bolger A.F., Engvall J., Carlhäll C.J. (2016). Altered Diastolic Flow Patterns and Kinetic Energy in Subtle Left Ventricular Remodeling and Dysfunction Detected by 4D Flow MRI. PLoS ONE.

[22] Zajac J., Eriksson J., Alehagen U., Ebbers T., Bolger A.F., Carlhäll C.-J. (2018). Mechanical dyssynchrony alters left ventricular flow energetics in failing hearts with LBBB: A 4D flow CMR pilot study. Int. J. Cardiovasc. Imaging.

[23] Eriksson J., Dyverfeldt P., Engvall J., Bolger A.F., Ebbers T., Carlhäll C.J. (2011). Quantification of presystolic blood flow organization and energetics in the human left ventricle. Am. J. Physiol. Heart Circ. Physiol..

[24] Al-Wakeel N., Fernandes J.F., Amiri A., Siniawski H., Goubergrits L., Berger F., Kuehne T. (2015). Hemodynamic and energetic aspects of the left ventricle in patients with mitral regurgitation before and after mitral valve surgery. J. Magn. Reason. Imaging.

