# Supplementary material for: Left Ventricular Blood Flow Kinetic Energy Assessment by 4D Flow Cardiovascular Magnetic Resonance: A Systematic Review of the Clinical Relevance
Source: J Cardiovasc Dev Dis. 2020 Sep 10;7(3):37. doi: 10.3390/jcdd7030037 (PMC7569817; doi:10.3390/jcdd7030037)
Supplement: Supplementary file 1 [file jcdd-07-00037-s001.pdf]

Supplementary Figure S1. Search details carried out on Medline and PubMed on 5th January 2019.

☐ 188	Medline	(left ventricle kinetic energy).ti,ab	View Results (46)
☐ 189	Medline	(left ventricle energetics).ti,ab	View Results (76)
☐ 190	Medline	(intra ventricular energetics).ti,ab	View Results (8)
☐ 191	Medline	(four dimensional flow energetics).ti,ab	View Results (5)
☐ 192	Medline	(4 D flow kinetic energy).ti,ab	View Results (32)
☐ 193	Medline	(4 D flow energetics).ti,ab	View Results (8)
☐ 194	Medline	(LV energetics).ti,ab	View Results (129)
☐ 195	Medline	(Cardiac Magnetic Resonance flow kinetics).ti,ab	View Results (11)
☐ 196	Medline	*(188 OR 189 OR 190 OR 191 OR 192 OR 193 OR 194 OR 195)*	View Results (281)
☐ 197	PubMed	(left ventricle kinetic energy).ti,ab	View Results (71)
☐ 198	PubMed	(left ventricle energetics).ti,ab	View Results (242)
☐ 199	PubMed	(intra ventricular energetics).ti,ab	View Results (3)
☐ 200	PubMed	(four dimensional flow energetics).ti,ab	View Results (2)
☐ 201	PubMed	(4 D flow kinetic energy).ti,ab	View Results (1)
☐ 202	PubMed	(4 D flow energetics).ti,ab	View Results (0)
☐ 203	PubMed	(LV energetics).ti,ab	View Results (14)
☐ 204	PubMed	(Cardiac Magnetic Resonance flow kinetics).ti,ab	View Results (4)
☐ 205	PubMed	*(197 OR 198 OR 199 OR 200 OR 201 OR 202 OR 203 OR 204)*	View Results (322)

Supplementary Table S1. Unique database specific exclusion criteria for the final search of relevant papers.

Medline	Pubmed	EMBASE	CINAHL
Echo	Animal study	AR	Cellular–ribose/ubiquinol
RV	PET scan study	Arterial Pulse waveform	Echo
Arterial measurements	Biologic engineering	Invasive catheterisation measurements	Review
Cardiac assisted devices	Right ventricle study	Echo measurements	Right ventricle

Severe AS	Russian, Japanese	Animal sample-mice, pigs, rabbits, sheep, dogs	LA measurements
Biomedical engineering	MRI results not related to LV	Biomedical engineering	MRA
Dynamic whole body study	Invasive measurements	Right ventricle	Animal -rabbit
Review article	Molecular and cellular cardiology		Pediatrics
LVAD, prosthetic valves	Surgical procedure-ventriculectomy		Pulmonary artery measurements
Pediatrics	Children		
Non cardiac MR modalities—PET	Atrial tissue, LVAD		

☐ 214	CINAHL	(left ventricle kinetic energy).ti,ab	View Results (10)
☐ 215	CINAHL	(left ventricle energetics).ti,ab	View Results (12)
☐ 216	CINAHL	(intra ventricular energetics).ti,ab	View Results (4)
☐ 217	CINAHL	(four dimensional flow energetics).ti,ab	View Results (4)
☐ 218	CINAHL	(4 D flow kinetic energy).ti,ab	View Results (0)
☐ 219	CINAHL	(4 D flow energetics).ti,ab	View Results (0)
☐ 220	CINAHL	(LV energetics).ti,ab	View Results (21)
☐ 221	CINAHL	(Cardiac Magnetic Resonance flow kinetics).ti,ab	View Results (11)
☐ 222	CINAHL	*(214 OR 215 OR 216 OR 217 OR 218 OR 219 OR 220 OR 221)*	View Results (49)
☐ 223	EMBASE	(left ventricle kinetic energy).ti,ab	View Results (0)
☐ 224	EMBASE	(left ventricle energetics).ti,ab	View Results (2)
☐ 225	EMBASE	(intra ventricular energetics).ti,ab	View Results (0)
☐ 226	EMBASE	(four dimensional flow energetics).ti,ab	View Results (0)
☐ 227	EMBASE	(4 D flow kinetic energy).ti,ab	View Results (0)
☐ 228	EMBASE	(4 D flow energetics).ti,ab	View Results (0)
☐ 229	EMBASE	(LV energetics).ti,ab	View Results (23)
☐ 230	EMBASE	(Cardiac Magnetic Resonance flow kinetics).ti,ab	View Results (0)
☐ 231	EMBASE	*(223 OR 224 OR 225 OR 226 OR 227 OR 228 OR 229 OR 230)*	View Results (25)
